# Dependence of premature ventricular complexes on heart rate—it’s not that simple

**DOI:** 10.1093/jamia/ocaf069

**Published:** 2025-05-12

**Authors:** Adrien Osakwe, Noah Wightman, Marc W Deyell, Zachary Laksman, Alvin Shrier, Gil Bub, Leon Glass, Thomas M Bury

**Affiliations:** Quantitative Life Sciences Program, McGill University, Montreal, QC H3A 1E3, Canada; Quantitative Life Sciences Program, McGill University, Montreal, QC H3A 1E3, Canada; Division of Cardiology and Centre for Cardiovascular Innovation, University of British Columbia, Vancouver, BC V6E 1M7, Canada; Division of Cardiology and Centre for Cardiovascular Innovation, University of British Columbia, Vancouver, BC V6E 1M7, Canada; Department of Physiology, McGill University, Montreal, QC H3G 1Y6, Canada; Department of Physiology, McGill University, Montreal, QC H3G 1Y6, Canada; Department of Physiology, McGill University, Montreal, QC H3G 1Y6, Canada; Department of Physiology, McGill University, Montreal, QC H3G 1Y6, Canada

**Keywords:** cardiac arrhythmia, premature ventricular complexes, Holter monitoring, classification, wearables

## Abstract

**Objective:**

Frequent premature ventricular complexes (PVCs) can lead to adverse health conditions such as cardiomyopathy. The linear correlation between PVC frequency and heart rate (as positive, negative, or neutral) on a 24-hour Holter recording has been proposed as a way to classify patients and guide treatment with beta-blockers. Our objective was to evaluate the robustness of this classification to measurement methodology, different 24-hour periods, and nonlinear dependencies of PVCs on heart rate.

**Materials and Methods:**

We analyzed 82 multi-day Holter recordings (1-7 days) collected from 48 patients with frequent PVCs (burden 1%-44%). For each record, linear correlation between PVC frequency and heart rate was computed for different 24-hour periods and using different length intervals to determine PVC frequency.

**Results:**

Using a 1-hour interval, the correlation between PVC frequency and heart rate was consistently positive, negative, or neutral on different days in only 36.6% of patients. Using shorter time intervals, the correlation was consistent in 56.1% of patients. Shorter time intervals revealed nonlinear and piecewise linear relationships between PVC frequency and heart rate in many patients.

**Discussion:**

The variability of the correlation between PVC frequency and heart rate across different 24-hour periods and interval durations suggests that the relationship is neither strictly linear nor stationary. A better understanding of the mechanism driving the PVCs, combined with computational and biological models that represent these mechanisms, may provide insight into the observed nonlinear behavior and guide more robust classification strategies.

**Conclusion:**

Linear correlation as a tool to classify patients with frequent PVCs should be used with caution. It is sensitive to the specific 24-hour period analyzed and the methodology used to segment the data. More sophisticated classification approaches that can capture nonlinear and time-varying dependencies should be developed and considered in clinical practice.

## Introduction

Premature ventricular complexes (PVCs) are a common finding in otherwise healthy patients.^1^ If PVCs are frequent, there is a risk of progression onto heart failure and the triggering of more serious arrhythmia.^2^^,^^3^ In a study of patients with frequent PVCs, Winkle found that most patients displayed characteristic relationships between PVC frequency and heart rate. Most frequently, there was a positive correlation (as assessed visually) in which there was a greater frequency of PVCs at faster heart rates, but in some patients, there could be a negative correlation, no correlation, or more complex relationships.^4^ Winkle suggested that the majority of patients expressing a positive correlation may help explain why most patients have a reduction in PVC frequency when treated with beta-blocking agents. The relationship of fewer PVCs at lower heart rates might also explain the well-known phenomenon of sleep suppression of PVCs in some patients.

Consistent with these early results, Hamon et al found that a positive correlation between PVC frequency and heart rate is useful to select patients who will respond to beta-blocker therapy.^5^ However, another study showed a conservative approach can be just as effective at reducing PVCs and found no differential effect of beta-blockers based on heart rate dependency.^6^ Hamon et al also found that patients with a positive correlation between PVC frequency and heart rate had a higher success rate to ablation therapy than patients with other characteristic relationships.^7^ In carrying out their analyses, Hamon et al considered the dependence by computing the Pearson correlation coefficient based on a 24-hour Holter recording in which the average PVC frequency and heart rate were determined over hour-long intervals. These findings have recently been included in the European Society for Cardiology’s guidelines for managing patients with frequent PVCs.^8^

Since the heart rate shows significant fluctuation on a time scale of minutes,^9^ subtle dependencies of PVC frequency on heart rate might be obscured by considering the average heart rate and PVC frequency over hour long intervals. In fact, Winkle’s initial study considered the average heart rate and PVC frequency over 1-minute rather than 1-hour intervals. The effect of this interval duration on the PVC-HR correlation has not been investigated. In addition, we have access to multi-day recordings that permit an analysis of the variability of a patient’s PVC-HR correlation over several days. Winkle found that the PVC-HR relationship was reproducible in 21 out of 24 patients in follow-up 24-hour recordings taken between 1 day and 2 months after the initial recording.

In the current article, we investigate the robustness of the PVC-HR correlation to (i) the methodology used to compute PVC frequency and heart rate and (ii) different 24-hour periods in multi-day records. Methodologies include using different time intervals over which to count the PVCs, and whether or not to aggregate the time intervals by heart rate when computing a PVC frequency. We also determine how specific PVC rhythms such as bigeminy (alternating PVCs and regular beats) influence the overall dependence of PVC frequency on heart rate.

## Methods

### Data collection and pre-processing

We collected 82 ECG recordings of 1-7 days from 48 patients with idiopathic frequent PVCs using a wearable device (Icentia CardioSTAT) at the University of British Columbia.^10^ Beat annotations were generated using Icentia’s proprietary software and subsequently reviewed by a team of technologists. Any sections of the recordings obscured by noise were excluded from the analysis. Specifically, the recordings were divided into non-overlapping 30-second segments, and segments with noise were discarded.

**Figure 1.**
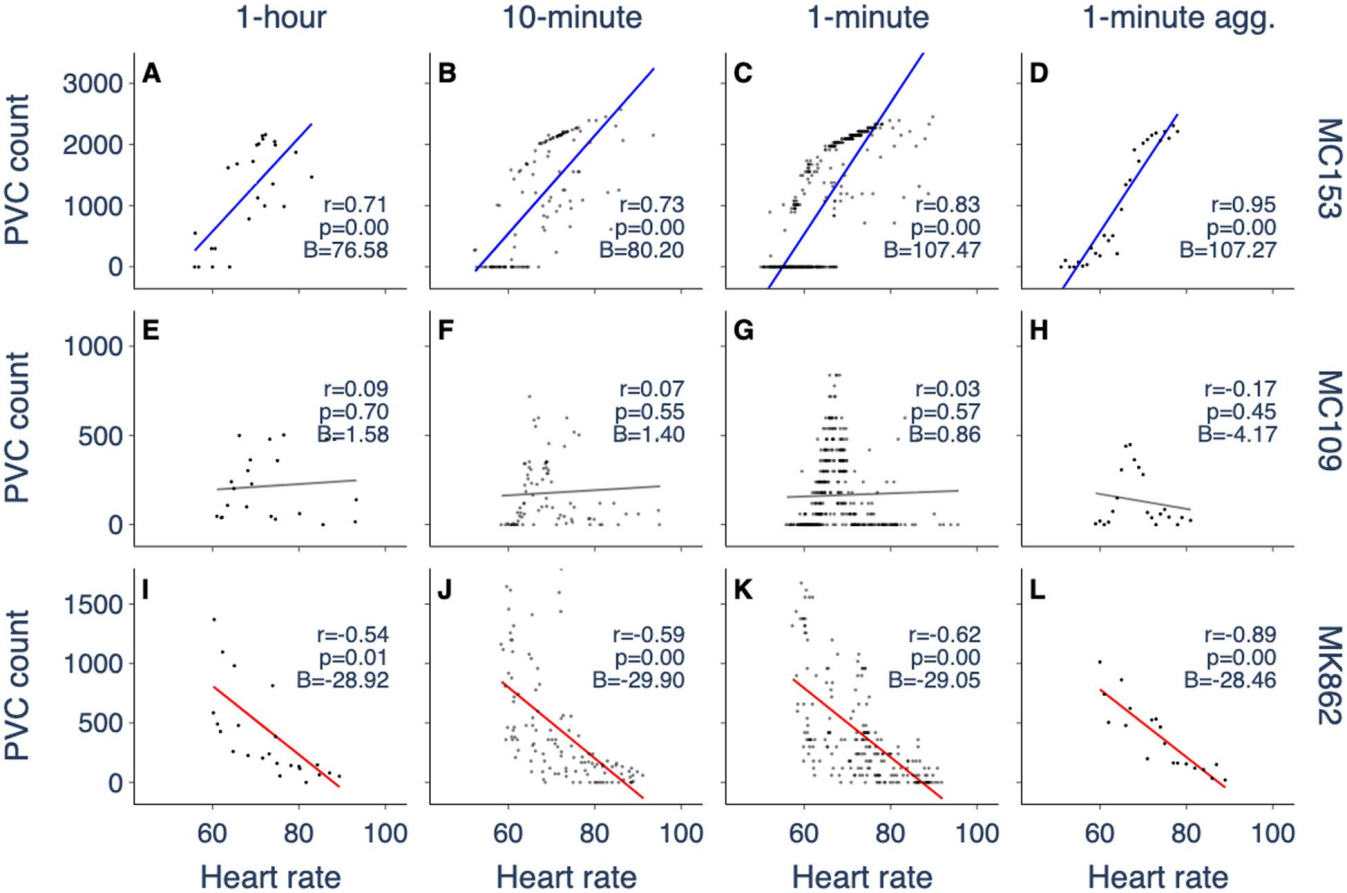
PVC-HR relationship for 3 different patients over a 24-hour period computed using different time interval durations. Rows show individual patients with positive (A-D), neutral (E-H), and negative (I-L) classifications. Columns show the PVC-HR relationship using 1-hour, 10-minute, and 1-minute time intervals, and 1-minute time intervals aggregated by heart rate. PVC count is scaled to the expected number of PVCs in 1 hour to facilitate comparison across methodologies. Lines show the linear regression. Correlation of PVC count with heart rate is positive (blue), neutral (gray, P>.05), or negative (red). Inset shows Pearson’s correlation coefficient (*r*), *P*-value (*P*), and the slope of the linear regression (*B*). The PVC-HR relationship on a log-linear scale is shown in Figure S1.

We computed the average heart rate and the total number of PVCs for interval durations of 1 hour, 10 minutes, and 1 minute. We visualize the PVC-HR relationship as a scatter plot, where each point represents a time interval, and the PVC count is scaled up to represent the expected number of PVCs in 1 hour (Figure 1, left 3 columns). To facilitate comparison with Winkle’s study,^4^ we also consider a method where the minute intervals in the record are aggregated based on heart rate with 1 bpm spacing. The PVC count is computed if there are more than 10 distinct 1-minute intervals at a given heart rate, and scaled to the expected number of PVCs in 1 hour. This results in at most one data point for each heart rate (Figure 1, right column). We perform the same computations for the PVC-HR relationship on a log-linear scale (Supplementary Material).

The cohort had a mean age of 63.5 years (range 32-91) with 37.5% females. The PVC burden (number of PVCs over total number of beats) at the time of the 7-day patch monitor was a median of 14.5% (range 1%-44%). The mean left ventricular ejection fraction (LVEF) was 52.3% (range 25%-72%) with 16 out of 48 (33.3%) having an LVEF of <50%. Of these 16 patients with impaired LV function, 14 (29.17%) had non-ischemic left ventricular dysfunction, 1 (2.1%) had an ischemic cardiomyopathy, and 1 (2.1%) had a mixed valvular and non-ischemic cardiomyopathy. 60.4% of patients were taking antiarrhythmic medication of some form.

### Statistical analysis

We computed the Pearson correlation coefficient and linear regression of PVC count against heart rate. The Pearson correlation coefficient is a measure of linear association between the 2 variables, computed as


(1) $$r=\frac{\text{Cov}(X,Y)}{\sigma_{X}\sigma_{Y}},$$

where Cov(X,Y) is the covariance between the heart rate (*X*) and the PVC count (*Y*), and σ is the standard deviation. A *P*-value is obtained using the Wald Test with a *t*-distribution of the test statistic. Linear regression determines the line of best fit between the variables. We used the ordinary least squares solution, with slope


(2) $$B=\frac{\text{Cov}(X,Y)}{\sigma_{X}^{2}}.$$

Each patient was classified as having a positive (r>0, P<.05), negative (r<0, p<.05), or neutral correlation (P>.05) as in Hamon et al.^5^^,^^7^ Classifications were made for each 24-hour section and for the whole recording. We also compute classifications using a log-linear scale for comparison with the study by Winkle.^4^ Specifically, the linear regression is conducted on the base-10 logarithm of the PVC count. Intervals with no PVCs are not defined on this scale and are therefore not plotted.

Variability of the classification to different days was quantified using Shannon entropy, which is a measure of the uncertainty of a source of information. It is computed as


(3) $$H(j)=-\sum_{c_{j}\in C}p(c_{j}){\;\text{log}\;}_{2}p(c_{j}),$$

where $p(c_{j})$ is the proportion of days for which a patient *j* was assigned class $c_{j}$ and C is the set of all possible classes (positive, negative, and neutral). We normalized the entropy to be between 0 and 1. A value of 0 indicates a consistent classification on each day whereas higher values indicate a more variable set of classifications. For a 7-day record, the highest entropy would be obtained if each of the 3 classes were assigned with a frequency of 2 days, 2 days and 3 days, respectively.

In addition to the average heart rate and PVC count, we determined the PVC rhythms, as defined by the number of intervening regular beats (NIBs) between consecutive PVCs. For example, an NIB of 1 corresponds to bigeminy (alternating regular beats and PVCs), an NIB of 2 corresponds to trigeminy, etc. We computed the NIB values for each 1-minute interval in the records.

All processing and analysis scripts are publicly available at the GitHub repository https://github.com/aosakwe/PVC-HR.

## Results

### Impact of time interval duration

Recent studies that evaluate the dependence of PVC frequency on heart rate use a 1-hour time interval to compute the PVC count and average heart rate.^5^^,^^7^^,^^10–13^ We show this approach for 3 patients that fall into the positive, neutral, and negative classes, respectively (Figure 1, first column). Using shorter time intervals (Figure 1, second and third columns) yields more data points and a wider distribution of PVC counts and heart rates. This is expected as shorter time intervals capture brief moments of particularly fast or slow heart rates that would otherwise be averaged out. Observing PVC count with a shorter time interval reveals (i) regions of high density with structure (eg, piece-wise linear trends in Figure 1C) and (ii) nonlinearities (eg, the hump-like structure in Figure 1G). These observations challenge the feasibility of using linear correlation as a classification metric. Using 1-minute intervals aggregated by heart rate (Figure 1, fourth column) captures linear and nonlinear trends, but averages out regions where PVC count can differ at a given heart rate, such as in Figure 1C.

Shortening the time interval duration can result in a different classification for the same patient. This can occur due to the additional data points at faster/slower heart rates altering the Pearson correlation or, more commonly, the additional data points reducing the *P*-value and causing a neutral classification to become positive or negative. In general, we find that shorter time intervals reduce the number of neutral classifications in the cohort (Table 1).

**Table 1.** Proportion of positive, negative, and neutral classifications across the cohort of 82 patients, grouped by day and methodology.

	Day							
Method	Classification	0	1	2	3	4	5	6
**1 hr**	**Positive**	0.65	0.57	0.62	0.53	0.57	0.59	0.63
	**Neutral**	0.29	0.38	0.33	0.42	0.34	0.38	0.31
	**Negative**	0.06	0.05	0.05	0.05	0.09	0.03	0.06
**10 min**	**Positive**	0.77	0.75	0.74	0.68	0.73	0.75	0.71
	**Neutral**	0.18	0.15	0.17	0.21	0.14	0.17	0.20
	**Negative**	0.05	0.10	0.09	0.11	0.14	0.07	0.09
**1 min**	**Positive**	0.67	0.74	0.71	0.67	0.70	0.78	0.57
	**Neutral**	0.13	0.05	0.11	0.09	0.11	0.06	0.19
	**Negative**	0.20	0.21	0.18	0.24	0.19	0.16	0.24
**1 min agg.**	**Positive**	0.60	0.61	0.61	0.59	0.62	0.65	0.57
	**Neutral**	0.29	0.22	0.25	0.21	0.23	0.22	0.32
	**Negative**	0.11	0.16	0.14	0.20	0.15	0.13	0.11

### Variability across different 24-hour periods

**Figure 2.**
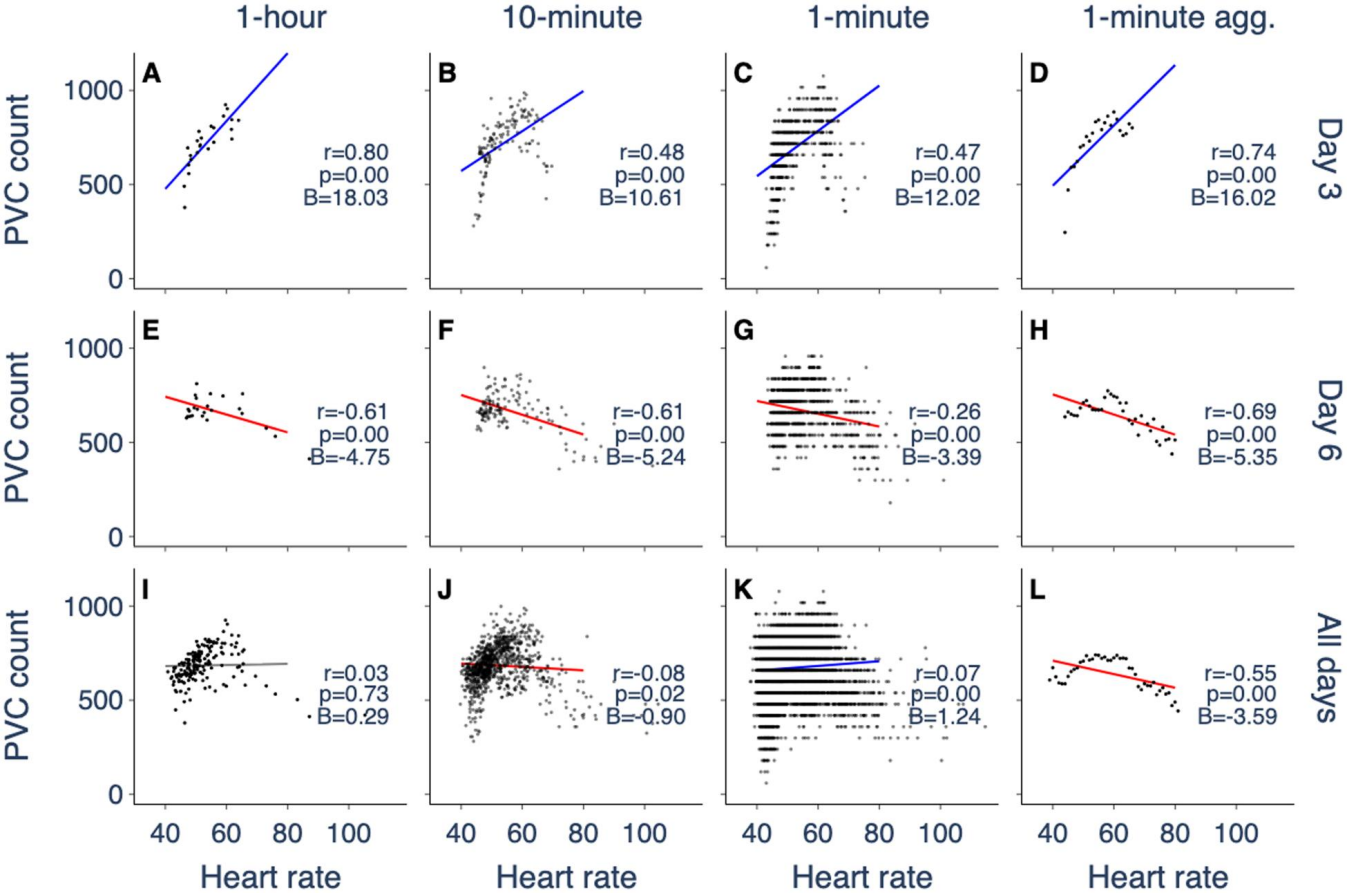
Patients with complex PVC-HR relationships show variable linear classifications across different 24-hour periods and different methodologies. Panels show the PVC-HR relationship for a single patient (MK993) computed using the third day (A-D), sixth day (E-H), and all days (I-L) of the recording. This patient shows a nonlinear PVC-HR relationship with highest PVC frequency at intermediate heart rates. The PVC-HR relationship on a log-linear scale is shown in Figure S2. Further details are in the caption of Figure 1.

Previous studies have assessed PVC-HR correlation using 24-hour Holter records,^4^^,^^5^^,^^7^^,^^11–13^ with some studies reporting PVC-HR correlation in follow-up recordings.^4^ In contrast, our dataset includes recordings of up to 7 days, allowing us to assess PVC-HR correlation on several different days. Figure 2 shows a patient who receives different classifications for different 24-hour periods, regardless of the methodology used. On the third day, they receive a positive classification (Figure 2A-D) whereas on the sixth day, they receive a negative classification (Figure 2E-H). Their PVC-HR relationship over the entire record (Figure 2I-L) resembles an inverted parabola. The discrepancy in classifications on different days may therefore be due to different heart rate distributions on different days, resulting in different sections of the PVC-HR relationship being captured.

**Figure 3.**
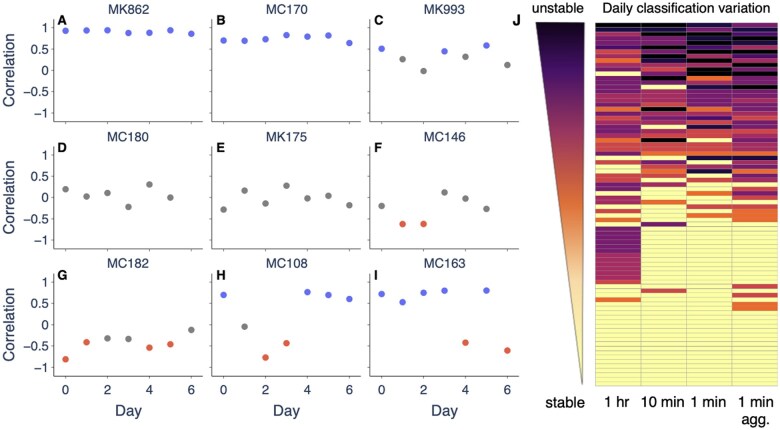
Daily variability of PVC-HR classification. (A-I) Pearson correlation coefficient and PVC-HR classification as positive (blue), negative (red), or neutral (gray) on different days for a sample of 9 patients using 1-hour intervals. (J) Variation in PVC-HR classification for each record (row) using different methodologies (columns). Variation is quantified using (normalized) entropy which goes from 0 (yellow) to 1 (black). Patients with low entropy have a consistent classification (eg, A). Patients with high entropy have an inconsistent classification (eg, C). Classifications using a log-linear scale are shown in Figure S3.

We quantify daily classification variation in terms of entropy (Figure 3). Zero entropy corresponds to a consistent classification each day, whereas a non-zero entropy corresponds to an inconsistent classification. We find that patients can jump between 2 or, in rare cases, all 3 of the classifications over the week. Using 1-hour, 10-minute, 1-minute, and aggregated 1-minute time intervals, the proportion of patients with zero entropy (ie, the same classification on each day) is 36.6%, 56.1%, 56.1% and 42.7%, respectively. Using a log-linear scale, the proportions are 40.2%, 47.6%, 47.6% and 43.9%. The method that yields the lowest median entropy across the cohort is using 1-minute or 10-minute intervals (not aggregated) on a linear scale (Figure S4).

In Winkle’s study, 21 out of 24 patients (87.5%) exhibited a consistent PVC-HR relationship across 2, 24-hour Holter recordings taken 1 day to 2 months apart. This finding appears to contrast with our results. However, our measure of consistency spans up to 7, 24-hour sections of a multi-day Holter recording. For a more direct comparison with Winkle’s result, we computed the probability that 2 randomly selected 24-hour sections from a given patient would yield the same classification (using 1-minute aggregated intervals and a log-linear scale, as in Winkle’s study). This yields an expectation of 67.7% of the patients receiving 2 identical classifications—lower than Winkle’s reported 87.5%. This discrepancy likely arises from differences in the methods used for classification. Winkle used visual inspection, while we used the method by Hamon et al, which uses linear regression. While these approaches may yield similar classifications for patients with a strong positive or negative correlation, they may differ for patients with more subtle or complex PVC-HR relationships. While visual inspection is useful in small studies, it would be impractical for large datasets and would introduce variability across physicians and patient cohorts.

**Figure 4.**
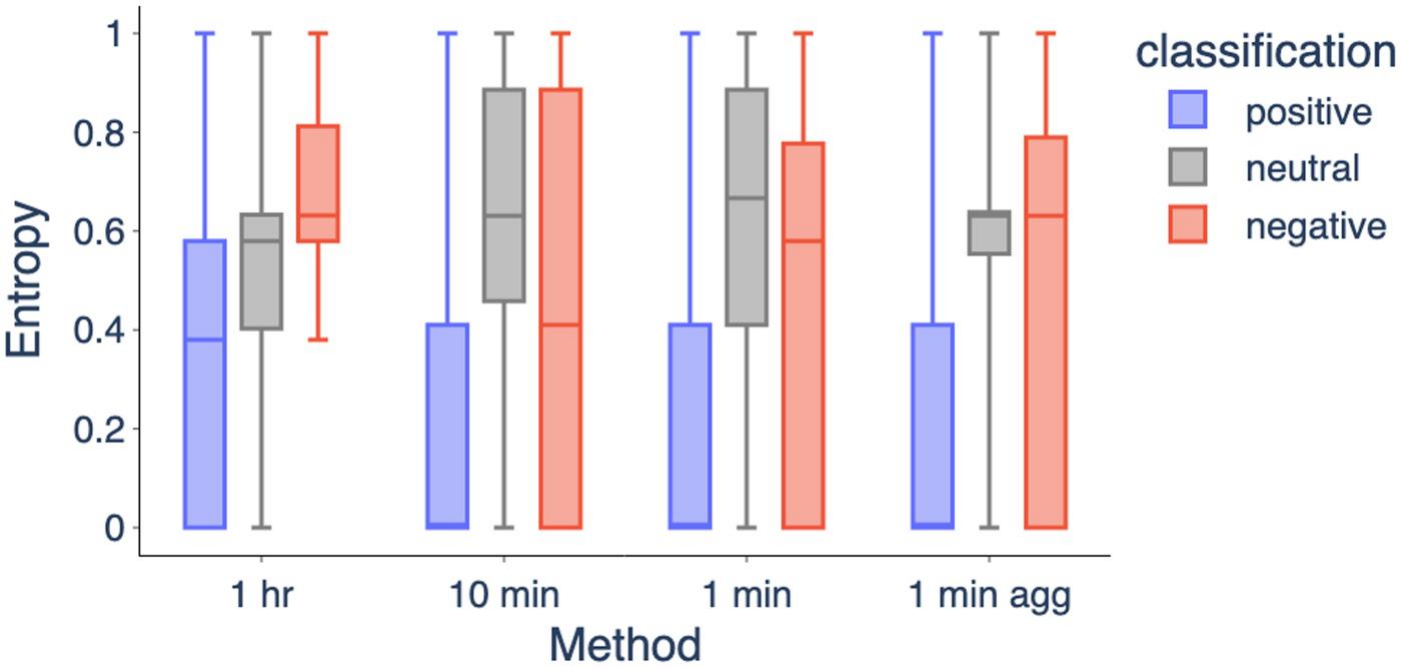
Entropy of PVC-HR classifications as a function of methodology and class. Patients with a positive classification on a given day typically have lower entropy scores (the same classification on the other days). Box center line is the median, box edges are the upper and lower quartiles, and whiskers capture the range up to 1.5 times the interquartile range.

For each 24-hour classification, we plot the entropy of the corresponding set of 24-hour classifications assigned to the patient in Figure 4. We find that patients with a positive classification on a given day tend to have lower entropy scores, ie, the classification is more consistent. Patients with a negative or a neutral classification are more likely to jump between different classes over the week.

**Figure 5.**
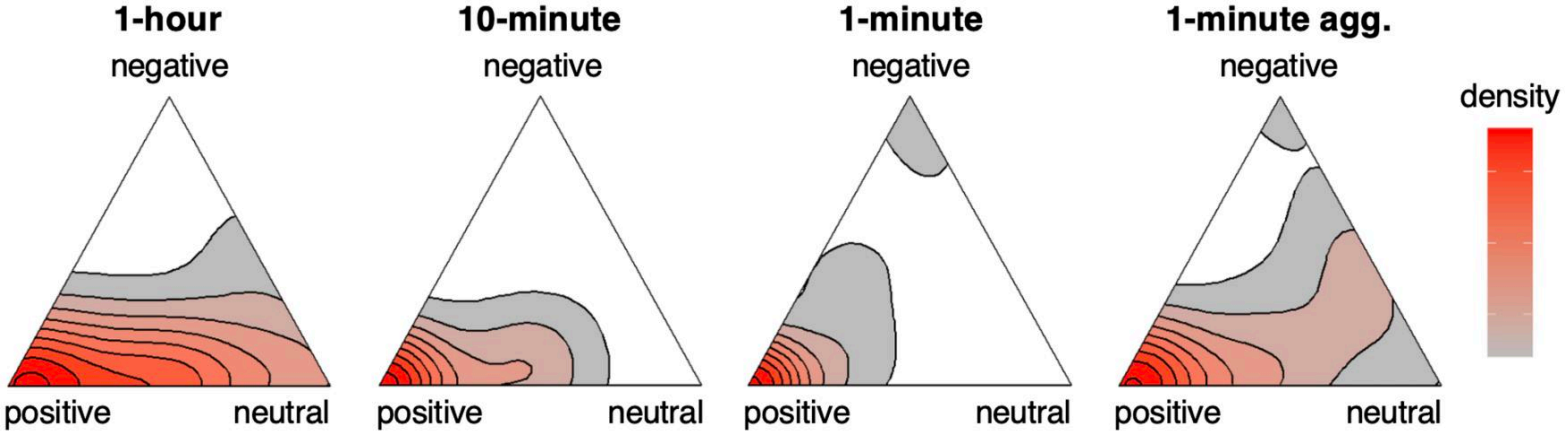
Distribution of classification sets for each patient using different methodologies. A classification set contains each 24-hour classification of a patient and is positioned on the triangle according to the ratios between each classification frequency. A patient with the same classification on each day would be placed in a corner. Records with different classifications on each day are placed in the interior according to the relative frequencies of each classification. The distribution is shown using a ternary density plot with red (white) representing a high (low) concentration of patients. The distributions for classifications using a log-linear scale are shown in Figure S5.

The set of daily classifications for each patient can be viewed as a point on a ternary plot, which graphically depicts the ratios of positive, negative, and neutral classifications. Figure 5 shows the distribution of these points for the cohort. Using a 1-hour interval, most patients receive a combination of positive and neutral classifications over the week. Shortening to a 10-minute interval, there are far fewer patients with a consistent neutral classification. Shortening still to a 1-minute interval, positive and negative classifications become more consistent.

### Stable rhythms in the PVC-HR relationship

**Figure 6.**
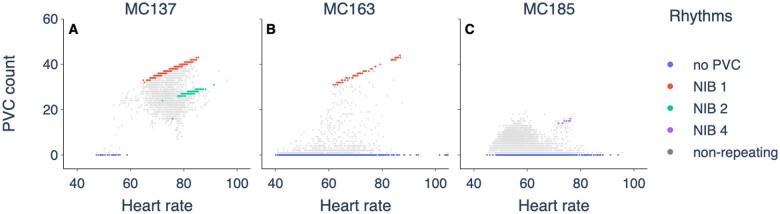
PVC count against heart rate for records with a positive (A, B) and negative (C) classification using 1-minute intervals. Intervals with a repeating rhythm are colored. Intervals without a repeating rhythm are gray and transparent. Repeating rhythms include no PVCs, and a fixed number of intervening regular beats (NIB) between PVCs.

In many patients, we observe stable rhythms in the form of a fixed number of intervening regular beats (NIB) between PVCs. How these rhythms depend on heart rate necessarily reflects an underlying mechanism for the PVCs. In Figure 6, we show the PVC count against heart rate for 1-minute intervals with colored points denoting intervals with a fixed NIB value. In record MC137 (Figure 6A), we observe stable rhythms of no PVCs, PVCs alternating with regular beats (bigeminy, NIB = 1) and PVCs intervened by 2 regular beats (trigeminy, NIB = 2). For this case, the rhythms occur at quite different heart rates—no PVCs at slow heart rates (HR < 60 bpm), bigeminy at intermediate-to-fast heart rates (65 < HR < 85 bpm), and trigeminy at fast heart rates (HR > 80 bpm). Transitions between different stable rhythms as heart rate varies can occur for an arrhythmia called modulated parasystole, where PVCs are generated by a ectopic pacemaker that is reset (modulated) by the regular heart beat.^10^^,^^14^^,^^15^

In contrast, record MC163 shows both no PVCs and bigeminy (NIB = 1) over a wide range of overlapping heart rates. For this patient, heart rate alone is not enough to determine the PVC dynamics—other variables besides heart rate must be changing across the record. The occurrence of stable bigeminy and no PVCs at the same heart rate may be a manifestation of the “rule of bigeminy” where a bistability exists between these rhythms.^16^^,^^17^ The occurrence of a PVC results in a longer cycle due to a skipped beat, favoring a subsequent PVC and a repeating rhythm.^16^^,^^17^ This is associated with the mechanisms of triggered activity and reentry.

We can write down an analytical expression for the relationship between the PVC count and heart rate for rhythms with a fixed NIB. Let *T* be the period of the regular heartbeat in seconds (so the heart rate HR=60/T). We assume that for every PVC, the following regular beat is blocked. (This is the case for most PVCs. In some cases the following regular beat is expressed, in which case the PVC called “interpolated.”) Then a PVC occurs every (NIB+1)T seconds, meaning the number of PVCs in 1 minute is


(4) $$\text{PVC}\;\text{count}\;\text{per}\;\text{minute}=\frac{60}{(\text{NIB}+1)t_{s}}=\frac{\text{HR}}{\text{NIB}+1},$$

an increasing linear function of heart rate with a smaller gradient at larger NIBs. Therefore, stable rhythms on their own contribute to a positive PVC-HR relationship. Stable rhythms in combination can contribute to a positive or negative PVC-HR relationship depending on the order in which they appear and disappear as a function of heart rate. In particular, higher NIBs and no PVCs at faster heart rates will contribute to a negative relationship, as seen in Figure 6C.

## Discussion

Although infrequent PVCs are very common and are usually benign, PVCs can act as triggers for serious arrhythmia such as ventricular tachycardia and ventricular fibrillation.^18^ Furthermore, patients with frequent PVCs sometimes develop heart failure.^3^ It has been suggested that characterization of the dependence of PVC occurrence on the heart rate may be useful to help identify both the mechanism of PVCs in a patient and also help in choice of appropriate therapies.^4^^,^^5^^,^^7^ In this paper, we revisited the problem of determining the dependence of PVC frequency on the heart rate.

We assessed the robustness of linear correlation between PVC frequency and heart rate. Using shorter time intervals to count the PVCs and compute the average heart rate increased the number of data points, resulting in a more significant Pearson correlation coefficient and fewer patients being classified as neutral. It also revealed PVC frequencies at more extreme heart rates, which usually did not conform to a linear trend. The majority of patients did not receive the same classification on each day when using a 1-hour interval. This may provide partial reason for the conflicting reports on the efficacy of beta-blockers for treating PVCs.^5^^,^^6^ Using a shorter time interval improved consistency and revealed nonlinear and nonstationary aspects of the relationship between PVC frequency and heart rate.

An apparent contradiction is that shorter time intervals reveal nonlinear properties of the PVC-HR relationship, yet result in a more significant Pearson correlation coefficient (smaller *P*-value). This may be understood by the fact that shorter time intervals generate a larger number of data points, and most of these data lie within a short range of heart rates where the data are approximately linear. An interesting avenue for future research is to evaluate the PVC-HR relationship during exercise tests where a larger portion of the data come from faster heart rates.

The dependence of PVC frequency on heart rate necessarily reflects the underlying mechanism for the PVCs. For example, for pure parasystole, in which a protected ventricular pacemaker competes with the sinus rhythm, the number of PVCs at any heart rate is proportional to the fraction of time that the ventricle is not refractory.^15^ This fraction is greater at slower heart rates, resulting in a negative linear correlation between the frequency of PVCs and heart rate.^19^ In contrast, for situations in which the parasystolic focus is reset by the regular heartbeat, more complex relationships between heart rate and PVC frequency occur since heart rates at which no PVCs occur can be sandwiched between heart rates that generate frequent PVCs.^14^^,^^19^^,^^20^ Reentry and triggered activity are mechanisms that are associated with the “rule of bigeminy,” where a bigeminal rhythm of alternating regular beats with PVCs is self-perpetuating. A patient with persistent bigeminy over a range of heart rates would have a linear dependence of PVC frequency on heart rate with a slope of 0.5.

Determining the mechanism of PVCs from observation of their dynamics is a challenging inverse problem where insights can be gained from mathematical^14^^,^^19^^,^^21^ and biological^20^^,^^22^ models. Although most mathematical models of PVC dynamics consider heart rate as a governing parameter for changes in PVC dynamics,^10^^,^^14^^,^^20^ in the current work, we find that qualitatively different dynamics can occur at the same heart rate. This nonstationarity is consistent with the known dependence of PVC frequency on autonomic tone,^23^^,^^24^ exercise,^25^ caffeine intake,^26^ and the time of day.^10^

The PVC-HR relationship has been proposed as a method to guide beta-blocker treatment in patients with frequent PVCs^5^ and was recently included in the European Society of Cardiology (ESC) guidelines,^8^ highlighting its relevance to clinical decision-making. However, the PVC-HR relationship is not typically reported in contemporary analyses of ambulatory recordings, largely due to the uncertain clinical implications of such findings in relation to the pathophysiology of frequent PVCs. Developing more robust and nuanced methods beyond linear regression to classify the PVC-HR relationship could enable future studies to link these classifications with clinical outcomes, potentially improving patient management strategies.

Our analysis helps reveal potential roadblocks to a more complete understanding of PVC dynamics: (i) The PVC-HR relationship is not the same all the time in all patients. This indicates that there are additional factors, possibly involving environmental aspects such as activity and medications. Data sets will need to be expanded to include additional variables that affect PVC dynamics. (ii) For machine learning, large data sets will be needed. Such data sets potentially exist now in stored records from patients who have wearable devices that do beat recognition. However, these data are often not in a form suitable for research nor are they available for research. A recent step toward addressing this challenge is the Icentia11k dataset.^27^ (iii) Long-term outcome is not known for most patients. Cardiac disease evolves over many years. Although retrospective studies such as Do et al^18^ have succeeded in identifying critical factors of PVC dynamics implicated in serious disease, there is a need for long-term studies of massive data sets that include outcomes as well as transient factors such as timing of sleep, drugs (including coffee), and vigorous activity.

As originally observed by Winkle,^4^ the relationship between PVC frequency and heart rate is complex. This study brought together an interdisciplinary team of applied mathematicians and clinicians to evaluate the robustness of various methods for characterizing this relationship. This collaboration enabled the sharing of clinical data, the testing of methods to analyze the PVC-HR relationship, and the incorporation of clinical insights into the interpretation of the PVC-HR relationship. With the increasing number of ECGs recorded on wearable devices^28^ and the complexity of PVC rhythms in individual patients, continued interdisciplinary efforts will be crucial for developing data-driven classification methods for patients with frequent PVCs.

### Limitations

This is a retrospective study without data on clinical outcomes. No attempt was made to evaluate the correlation between PVC frequency and heart rate for each PVC morphology. Interpolated PVCs can result in overestimation of the heart rate, though were very uncommon. Finally, some records had regions of unidentifiable beats due to noise or artifact, which limited the amount of data available. Future analyses will benefit from improvements to wearable device technology and beat identification algorithms.

## Conclusion

The dependence of PVC frequency on heart rate is often nonlinear, can vary on different days, and can vary based on the duration of the time interval used in its computation. Therefore, linear correlation between PVC frequency and heart rate as a means to determine treatment groups should be used with caution. Classification of PVC dynamics should benefit from the massive collection of multi-day records and consideration of nonlinear (and/or piecewise linear) relationships, which should be enabled by interdisciplinary collaboration between the mathematical and cardiac sciences.

## Supplementary Material

ocaf069_Supplementary_Data

## Data Availability

This study used individual patient health data that cannot be shared without ethical approval. All processing and analysis scripts are publicly available at the GitHub repository https://github.com/aosakwe/PVC-HR.

## References

[1] Schamroth L. The Disorders of the Cardiac Rhythm. 2nd ed. Blackwell; 1980.

[2] Deyell MW , Hawkins NM. Odd couple: premature ventricular contractions and heart failure. Heart. 2022;108:86-87. 34667089 10.1136/heartjnl-2021-319986

[3] Marcus GM. Evaluation and management of premature ventricular complexes. Circulation. 2020;141:1404-1418. 32339046 10.1161/CIRCULATIONAHA.119.042434

[4] Winkle RA. The relationship between ventricular ectopic beat frequency and heart rate. Circulation. 1982;66:439-446. 7094251 10.1161/01.cir.66.2.439

[5] Hamon D , Swid MA, Rajendran PS, et al Premature ventricular contraction diurnal profiles predict distinct clinical characteristics and beta-blocker responses. J Cardiovasc Electrophysiol. 2019;30:836-843. 30964570 10.1111/jce.13944

[6] Tang JK , Andrade JG, Hawkins NM, et al Effectiveness of medical therapy for treatment of idiopathic frequent premature ventricular complexes. J Cardiovasc Electrophysiol. 2021;32:2246-2253. 34216056 10.1111/jce.15150

[7] Hamon D , Abehsira G, Gu K, et al Circadian variability patterns predict and guide premature ventricular contraction ablation procedural inducibility and outcomes. Heart Rhythm. 2018;15:99-106. 28765087 10.1016/j.hrthm.2017.07.034

[8] Zeppenfeld K , Tfelt-Hansen J, de Riva M, et al; ESC Scientific Document Group. 2022 ESC guidelines for the management of patients with ventricular arrhythmias and the prevention of sudden cardiac death: developed by the task force for the management of patients with ventricular arrhythmias and the prevention of sudden cardiac death of the European Society of Cardiology (ESC) endorsed by the Association for European Paediatric and Congenital Cardiology (AEPC). Eur Heart J. 2022;43:3997-4126.36017572

[9] Akselrod S , Gordon D, Ubel FA, Shannon DC, Berger AC, Cohen RJ. Power spectrum analysis of heart rate fluctuation: a quantitative probe of beat-to-beat cardiovascular control. Science. 1981;213:220-222. 6166045 10.1126/science.6166045

[10] Bury T , Lerma C, Bub G, Laksman Z, Deyell M, Glass L. Long ECGs reveal rich and robust dynamical regimes in patients with frequent ectopy. Chaos. 2020;30:113127. 33261339 10.1063/5.0023987

[11] Zhang B , Yu J, Wu Y, et al The significance of heart rate variability in patients with frequent premature ventricular complex originating from the ventricular outflow tract. Clin Cardiol. 2024;47:e24174. 37859500 10.1002/clc.24174 PMC10766131

[12] Demir S , Gulsen K, Kepez A, et al Predictors of positive response to beta-blockers for treatment of premature ventricular complexes. J Electrocardiol. 2022;70:50-55. 34922221 10.1016/j.jelectrocard.2021.11.036

[13] Serdar D , Kamil G, Abdulkadir U, et al Predictors of adequate intraprocedural premature ventricular complex (PVC) frequency during idiopathic PVC ablation. Herz. 2021;46:476. 33464357 10.1007/s00059-020-05017-8

[14] Moe G , Jalife J, Mueller W, Moe B. A mathematical model of parasystole and its application to clinical arrhythmias. Circulation. 1977;56:968-979. 923066 10.1161/01.cir.56.6.968

[15] Glass L , Goldberger AL, Bélair J. Dynamics of pure parasystole. Am J Physiol. 1986;251:H841-H847. 3766761 10.1152/ajpheart.1986.251.4.H841

[16] Lerma C , Lee CF, Glass L, Goldberger AL. The rule of bigeminy revisited: analysis in sudden cardiac death syndrome. J Electrocardiol. 2007;40:78-88. 17069837 10.1016/j.jelectrocard.2006.04.011

[17] Langendorf R , Pick A, Winternitz M. Mechanisms of intermittent ventricular bigeminy: I. Appearance of ectopic beats dependent upon length of the ventricular cycle, the “rule of bigeminy. Circulation. 1955;11:422-430. 14352386 10.1161/01.cir.11.3.422

[18] Do DH , O’Meara K, Lee J, et al Ventricular parasystole in cardiomyopathy patients: a link between His-Purkinje system damage and ventricular fibrillation, Clin Electrophysiol. 2023;9:936. 10.1016/j.jacep.2022.11.014 37438043

[19] Schulte-Frohlinde V , Ashkenazy Y, Goldberger AL, et al Complex patterns of abnormal heartbeats. Phys Rev E Stat Nonlin Soft Matter Phys. 2002;66:031901. 12366146 10.1103/PhysRevE.66.031901

[20] Antzelevitch C , Bernstein MJ, Feldman HN, Moe GK. Parasystole, reentry, and tachycardia: a canine preparation of cardiac arrhythmias occurring across inexcitable segments of tissue. Circulation. 1983;68:1101-1115. 6193902 10.1161/01.cir.68.5.1101

[21] Bury T , Diagne K, Olshan D, et al The inverse problem for cardiac arrhythmias. Chaos. 2023;33:123130. 38149994 10.1063/5.0161210

[22] Diagne K , Bury TM, Deyell MW, et al Rhythms from two competing periodic sources embedded in an excitable medium. Phys Rev Lett. 2023;130:028401. 36706395 10.1103/PhysRevLett.130.028401

[23] He W , Lu Z, Bao M, et al Autonomic involvement in idiopathic premature ventricular contractions. Clin Res Cardiol. 2013;102:361-370. 23386255 10.1007/s00392-013-0545-6

[24] Stein KM , Karagounis LA, Anderson JL, Kligfield P, Lerman BB. Fractal clustering of ventricular ectopy correlates with sympathetic tone preceding ectopic beats. Circulation. 1995;91:722-727. 7828299 10.1161/01.cir.91.3.722

[25] Lamb LE , Hiss RG. Influence of exercise on premature contractions. Am J Cardiol. 1962;10:209. 14461711 10.1016/0002-9149(62)90297-7

[26] Marcus GM , Rosenthal DG, Nah G, et al Acute effects of coffee consumption on health among ambulatory adults. N Engl J Med. 2023;388:1092-1100. 36947466 10.1056/NEJMoa2204737 PMC10167887

[27] Tan S , Ortiz-Gagné S, Beaudoin-Gagnon N, et al Icentia11k single lead continuous raw electrocardiogram dataset. *PhysioNet*, 2022. Accessed June 30, 2024. 10.13026/kk0v-r952

[28] Perez MV , Mahaffey KW, Hedlin H, et al; Apple Heart Study Investigators. Large-scale assessment of a smartwatch to identify atrial fibrillation. N Engl J Med. 2019;381:1909-1917. 31722151 10.1056/NEJMoa1901183 PMC8112605

