# Supplementary material for: Dependence of premature ventricular complexes on heart rate—it’s not that simple
Source: J Am Med Inform Assoc. 2025 May 12;33(1):90–7. doi: 10.1093/jamia/ocaf069 (PMC12758478; doi:10.1093/jamia/ocaf069)
Supplement: ocaf069_Supplementary_Data [file ocaf069_supplementary_data.zip › df_table1.docx]

Table 1: Proportion of positive, negative and neutral classifications across the cohort of 82 patients, grouped by day and methodology.

|  | **Day** | **0** | **1** | **2** | **3** | **4** | **5** | **6** |
| --- | --- | --- | --- | --- | --- | --- | --- | --- |
| **Method** | **Classification** |  |  |  |  |  |  |  |
| **1 hr** | **Positive** | 0.65 | 0.57 | 0.62 | 0.53 | 0.57 | 0.59 | 0.63 |
|  | **Neutral** | 0.29 | 0.38 | 0.33 | 0.42 | 0.34 | 0.38 | 0.31 |
|  | **Negative** | 0.06 | 0.05 | 0.05 | 0.05 | 0.09 | 0.03 | 0.06 |
| **10 min** | **Positive** | 0.77 | 0.75 | 0.74 | 0.68 | 0.73 | 0.75 | 0.71 |
|  | **Neutral** | 0.18 | 0.15 | 0.17 | 0.21 | 0.14 | 0.17 | 0.20 |
|  | **Negative** | 0.05 | 0.10 | 0.09 | 0.11 | 0.14 | 0.07 | 0.09 |
| **1 min** | **Positive** | 0.67 | 0.74 | 0.71 | 0.67 | 0.70 | 0.78 | 0.57 |
|  | **Neutral** | 0.13 | 0.05 | 0.11 | 0.09 | 0.11 | 0.06 | 0.19 |
|  | **Negative** | 0.20 | 0.21 | 0.18 | 0.24 | 0.19 | 0.16 | 0.24 |
| **1 min agg.** | **Positive** | 0.60 | 0.61 | 0.61 | 0.59 | 0.62 | 0.65 | 0.57 |
|  | **Neutral** | 0.29 | 0.22 | 0.25 | 0.21 | 0.23 | 0.22 | 0.32 |
|  | **Negative** | 0.11 | 0.16 | 0.14 | 0.20 | 0.15 | 0.13 | 0.11 |
