# Supplementary material for: Dependence of premature ventricular complexes on heart rate—it’s not that simple
Source: J Am Med Inform Assoc. 2025 May 12;33(1):90–7. doi: 10.1093/jamia/ocaf069 (PMC12758478; doi:10.1093/jamia/ocaf069)
Supplement: ocaf069_Supplementary_Data [file ocaf069_supplementary_data.zip › supplementary_info.pdf]

Dependence of premature ventricular complexes on heart rate —it's not that simple: Supplementary Information

Adrien Osakwe, BSc,[1] Noah Wightman, BSc,[1] Marc W. Deyell, MD,[2] Zachary Laksman, MD,[2] Alvin Shrier, PhD,[3] Gil Bub, PhD,[3] Leon Glass, PhD,[3] and Thomas M. Bury, PhD*[3]

[1] *Department of Quantitative Life Sciences, McGill University, 550 Sherbrooke W, Montreal, Quebec, H3A 1E3, Canada*

[2] *Division of Cardiology and Centre for Cardiovascular Innovation, University of British Columbia, Vancouver, British Columbia V6E 1M7, Canada*

[3] *Department of Physiology, McGill University, 3655 Promenade Sir William Osler, Montreal, Quebec H3G 1Y6, Canada*

(Dated: January 16, 2025)

*Corresponding author: Thomas Bury, PhD, Department of Physiology, 3655 Promenade Sir William Osler, Montréal, Québec, H3G 1Y6, Canada (thomas.bury@mcgill.ca)

Key words: cardiac arrhythmia, premature ventricular complexes, Holter monitoring, classification, wearables

		proportion						
	day	0	1	2	3	4	5	6
method	classification							
1hr	positive	0.66	0.54	0.62	0.57	0.57	0.62	0.57
	neutral	0.24	0.36	0.29	0.34	0.32	0.32	0.26
	negative	0.10	0.10	0.09	0.09	0.11	0.06	0.17
10min	positive	0.73	0.72	0.68	0.67	0.68	0.71	0.71
	neutral	0.17	0.20	0.22	0.18	0.18	0.22	0.17
	negative	0.10	0.08	0.09	0.14	0.15	0.07	0.11
1min	positive	0.62	0.68	0.70	0.66	0.61	0.71	0.57
	neutral	0.16	0.10	0.11	0.09	0.16	0.12	0.08
	negative	0.22	0.22	0.20	0.25	0.23	0.17	0.35
1min_agg	positive	0.61	0.61	0.61	0.62	0.66	0.70	0.59
	neutral	0.26	0.24	0.25	0.18	0.18	0.16	0.27
	negative	0.13	0.15	0.14	0.20	0.16	0.14	0.14

TABLE S1. Proportions of positive, negative and neutral classifications derived from linear regression of the log-linear plot for the cohort of 82 patients, grouped by day and methodology.

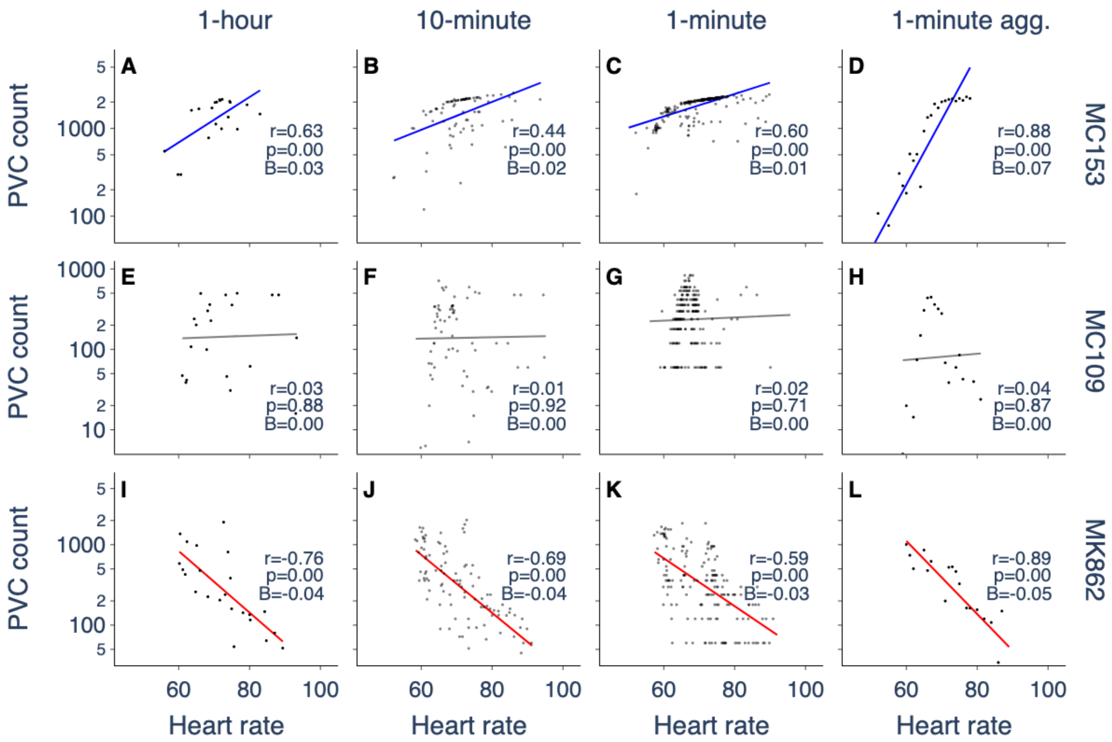

FIG. S1. PVC-HR relationship for three different patients (same as in Fig. 1) over a 24-hour period computed using different time interval durations on a log-linear scale. Rows show individual patients with positive (A-D), neutral (E-H) and negative (I-L) classifications. Columns show the PVC-HR relationship using 1-hour, 10-minute and 1-minute time intervals, and 1-minute time intervals aggregated by heart rate. PVC count is scaled to the expected number of PVCs in one hour to facilitate comparison across methodologies. Lines show the linear regression. Correlation of PVC count with heart rate is positive (blue), neutral (gray, $p > 0.05$) or negative (red). Inset shows Pearson's correlation coefficient (r), p-value (p) and the slope of the linear regression (B).

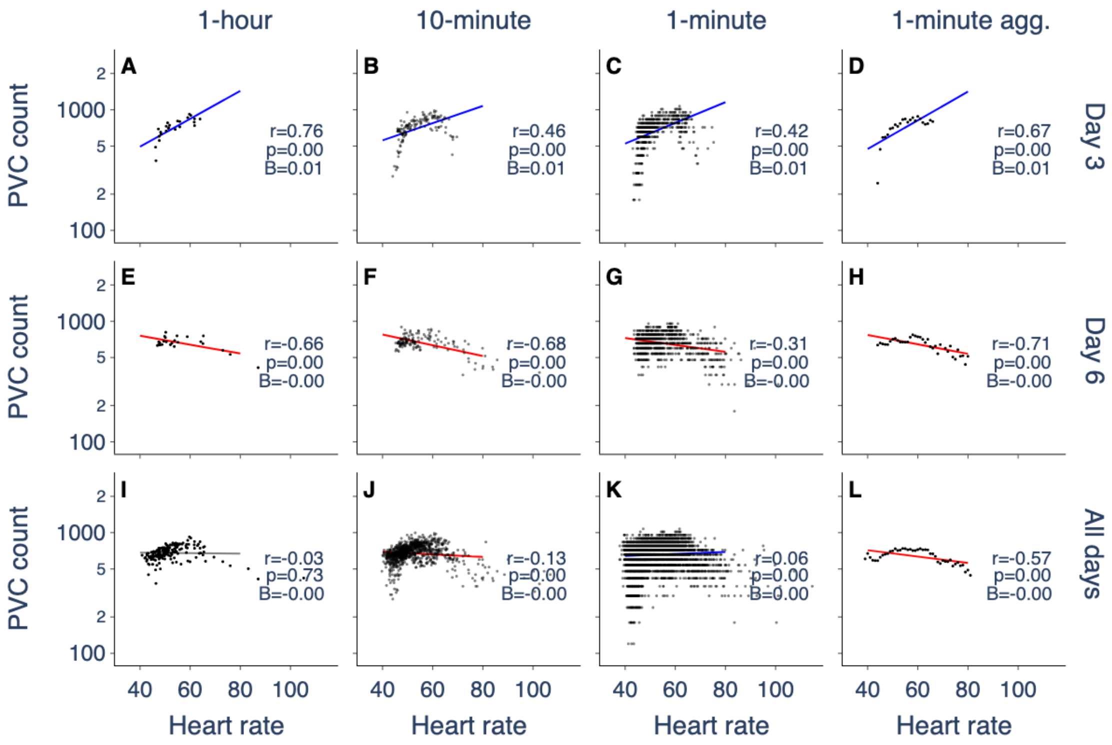

FIG. S2. PVC-HR relationship for a single patient (MK993) on a log-linear scale computed using the third day (A-D), sixth day (E-H) and all days (I-L) of the recording. Columns show the PVC-HR relationship using 1-hour, 10-minute and 1-minute time intervals, and 1-minute time intervals aggregated by heart rate. PVC count is scaled to the expected number of PVCs in one hour to facilitate comparison across methodologies. Lines show the regression on a log-linear scale. Correlation of PVC count with heart rate is positive (blue), neutral (gray, $p > 0.05$) or negative (red). Inset shows Pearson's correlation coefficient (r), p-value (p) and the slope of the linear regression (B).

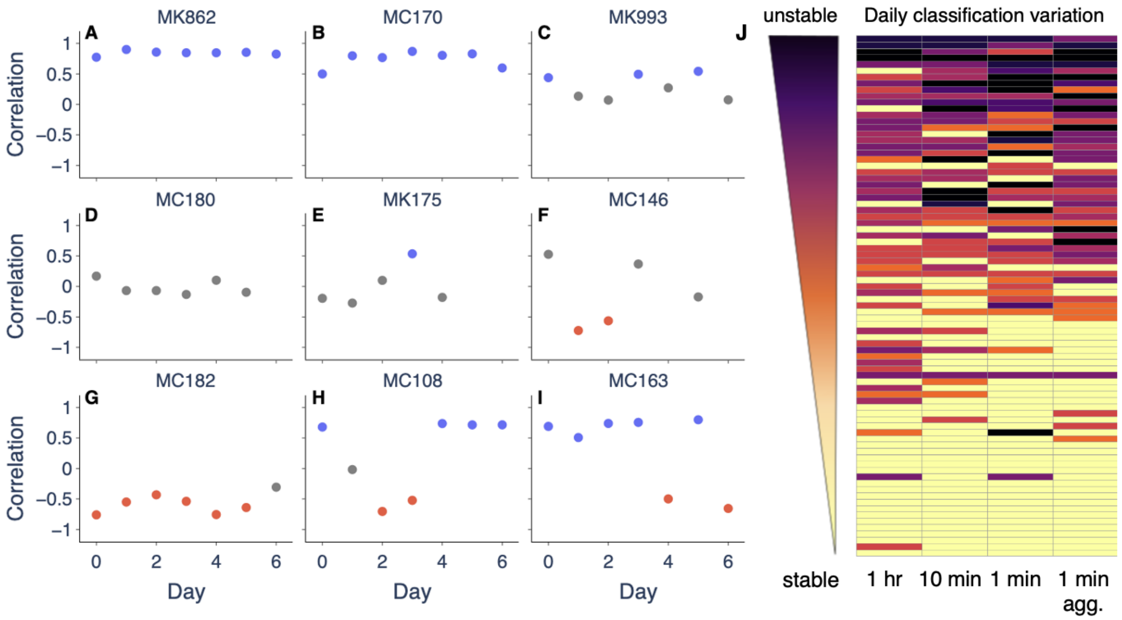

FIG. S3. Daily variability of PVC-HR classification on a log-linear scale. (A-I) Pearson correlation coefficient and PVC-HR classification as positive (blue), negative (red) or neutral (gray) on different days for a sample of 9 patients using 1 hour intervals. (J) Variation in PVC-HR classification for each record (row) using different methodologies (columns). Variation is quantified using (normalized) entropy which goes from zero (yellow) to one (black). Patients with low entropy have a consistent classification (e.g. A). Patients with high entropy have an inconsistent classification (e.g. I).

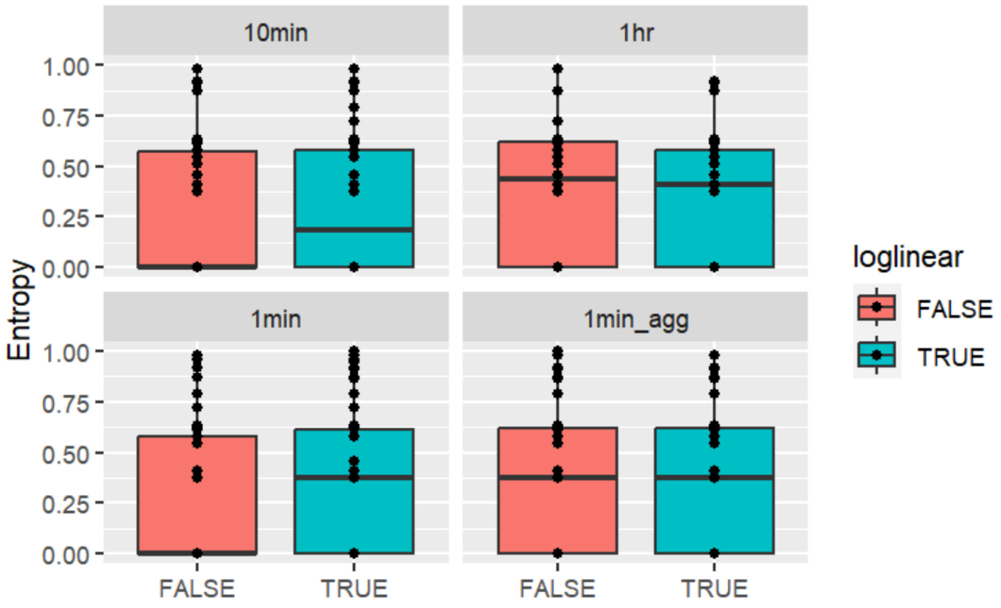

FIG. S4. Comparison of entropy scores across the 82 patients using different methodologies for computing the PVC-HR classification (see Methods section). The most consistent methodology is using 1-minute intervals to count PVCs and compute average heart rate and using a linear scale when computing the linear regression. Box center line is the median, box edges are the upper and lower quartiles, and whiskers capture the range up to 1.5 times the interquartile range.

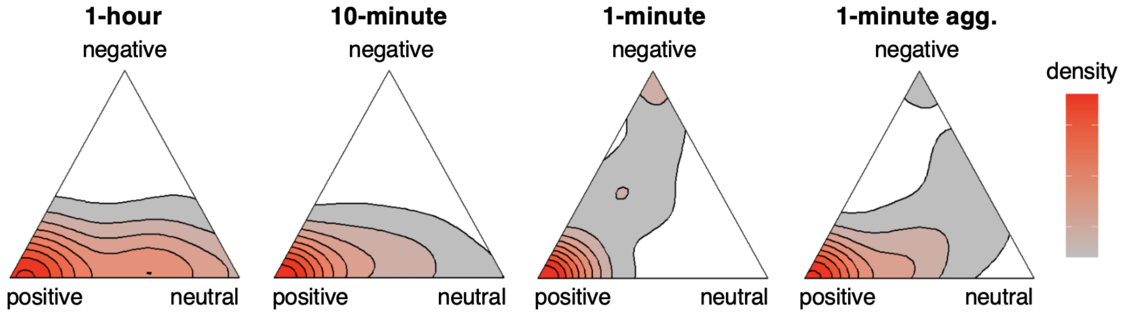

FIG. S5. Distribution of classification sets on a log-linear scale for each patient using different methodologies. A classification set contains each 24-hour classification of a patient on a log-linear scale and is positioned on the triangle according to the ratios between each classification frequency. A patient with the same classification on each day would be placed in a corner. Records with different classifications on each day are placed in the interior according to the relative frequencies of each classification. The distribution is shown using a ternary density plot with red (white) representing a high (low) concentration of patients.
